# Baicalin suppresses glaucoma pathogenesis by regulating the PI3K/AKT signaling in vitro and in vivo

**DOI:** 10.1080/21655979.2021.2001217

**Published:** 2021-12-03

**Authors:** Ningmin Zhao, Jieran Shi, Haohang Xu, Qing Luo, Qiaoyan Li, Mingzhou Liu

**Affiliations:** Department of Pharmacy, Henan Provincial People’s Hospital, Zhengzhou University People’s Hospital, Zhengzhou, China

**Keywords:** Baicalin, glaucoma, autophagy, oxidative stress, PI3K/AKT signaling

## Abstract

Glaucoma, characterized with progressive degeneration of retinal ganglion cells (RGCs), is the second frequently leading cause of sight loss in the word after cataract. Baicalin plays a protective role in age-related macular degeneration, retinopathy of prematurity, branch retinal vein occlusion, and ischemia-induced neurodegeneration in the retina. The present study aimed to investigate the role of baicalin in glaucoma. RGCs were stimulated with N-methyl-D-aspartate (NMDA) to mimic the *in vitro* model of glaucoma. A mouse model of glaucoma induced by chronic elevated intraocular pressure was also established. The apoptosis, oxidative stress, and autophagy of RGCs were detected by flow cytometry analysis, 2,7-dichlorodihydrofluorescein diacetate staining, and Western blotting, respectively. Retinal pathological changes were exhibited by hemotoxylin and eosin staining. Baicalin restrained the NMDA-induced cell apoptosis, autophagy, and oxidative stress of RGCs by activating the PI3K/AKT signaling *in vitro*. The elevated intraocular pressure-induced pathological changes in retinas of glaucoma mice were attenuated by baicalin. Moreover, the number of RGCs was significantly decreased in glaucoma mice, and then increased by baicalin treatment. Baicalin also inhibited autophagy and activated PI3K/AKT signaling *in vivo*. In conclusion, baicalin suppresses glaucoma pathogenesis by regulating the PI3K/AKT signaling *in vitro* and *in vivo*.

## Introduction

Glaucoma is a major cause of irreversible blindness around the world and is characterized by optic nerve injury and degeneration of the retinal ganglion cells (RGCs) [1]. Glaucoma is asymptomatic in the early stage, but leads to decline in contrast and color sensitivity and progressive visual field defects in its advanced stage [2]. In 2020, the number of people affected by glaucoma reached 4.5 million among global population with moderate or severe visual impairment [3]. The etiology of glaucoma is attributed to multiple factors, including oxidative stress, retinal ischemia, nutritional status, cardiovascular diseases, diabetes mellitus, glutamate excitotoxicity as well as hemorheological abnormalities [4,5]. Aqueous humor is the clear liquid filled in the anterior and posterior chambers of the eye, and obstruction of aqueous humor circulation can be caused by the elevated intraocular pressure (EIOP) [6]. As a receptor and protector of visual signals, RGCs play a crucial role in the transmission of visual information from the retina to the visual center of the brain [7]. EIOP easily contributes to RGC loss, stress response, and apoptosis of RGCs, subsequently causing pathological changes of glaucoma [8]. In addition, excessive autophagy and oxidative stress also can cause RGC apoptosis, indirectly leading to glaucoma [9,10]. Currently, the available approach for glaucoma treatment is to reduce IOP including surgical intervention, laser therapy, and medicine administration [11]. However, some anti-glaucoma drugs are associated with adverse symptons on eyes, including xerophthalmia, burning, and tingling sensation [12]. Therefore, identifying novel therapeutic strategies that can regulate RGC proliferation and IOP changes is important in glaucoma treatment and visual function recovery.

Baicalin is the dried root of *Scutellaria baicalensis Georgi*, a Chinese medicinal herb [13]. Baicalin can inhibit inflammation, oxidative stress, suppress tumor growth, and protect nerves [14]. Moreover, baicalin also can reduce serum cholesterol, capillary permeability, and blood pressure [15]. Thus, since ancient times, baicalin has been considered as a very popular drug in the clinical application of various diseases, such as hyperlipemia, leukemia, hepatoma, and hepatitis [16]. Multiple pharmacological properties of baicalin play important roles in the prevention and treatment of ocular diseases, such as glaucoma diabetic retinopathy age-related macular degeneration and cataract [17]. Baicalein exerts antioxidant and anti-apoptotic effects to prevent retinal ischemia by upregulating heme oxygenase-1 and downregulating hypoxia-inducible factor-1α, vascular endothelium growth factor, and matrix metalloproteinase 9 [18]. Baicalein is beneficial in cornea repair via inhibiting the production of platelet-activating factor [19]. Baicalin significantly inhibits the activity of aldose reductase (the culprit for diabetic cataract) in rat lens [20]. Baicalin dose-dependently exhibits protective effects against oxidative stress-induced RGC-5 injury, which indicates its beneficial role in preventing RGC loss in glaucoma [21]. Therefore, to further elucidate pharmacologic actions of baicalin in glaucoma progression, relevant studies should be conducted.

Based on the above studies, we made a hypothesis that baicalin exerts a protective role in glaucoma by alleviating RGC injury. The role and underlying pathway of baicalin in the apoptosis, autophagy, and oxidative stress of NMDA-stimulated RGCs and in pathological changes on retinas of glaucoma mice were investigated, which may provide more insight into the treatment of glaucoma.

## Materials and methods

### Cell culture

RGC-5 cells were purchased from Fenghui Biotechnology (Changsha, China). The cells were cultured in Dulbecco’s modified Eagle medium (DMEM; Invitrogen, USA) supplemented with 10% fetal bovine serum (Invitrogen), 100 μg/mL of streptomycin (Invitrogen) and 100 U/mL of penicillin (Invitrogen) at 37°C with 5% CO_2_. Medium was changed every 3 days and RGC-5 cells at passage of 3–5 were used.

### Cell treatment

NMDA at different concentrations (0, 50, 100, or 150 μmol/L) was used to treat RGC-5 cells for 30 min [22]. Cells were then cultured with the original culture medium for another 24 h. Since 150 μmol/L of N-methyl-D-aspartate (NMDA) has the best promotive effects on cell injury and autophagy of RGC-5 cells, NMDA at the concentration of 150 μmol/L was chosen to treat RGC-5 cells in the following experiments. Subsequently, RGC-5 cells were assigned into four groups. Cells in group I are basal cells without other treatments. Cells in group II were treated with 150 μmol/L of NMDA for 30 min. Cells in group III were treated with 150 μmol/L of NMDA for 30 min and 10 μmol/L of Baicalin (Sigma Aldrich, St. Louis, MO, USA) for 24 h [23]. 8-Phenyl-2-(morpholin-4-yl)-chromen-4-one (LY294002, formula: C₁₉H₁₇NO₃) is a broad-spectrum PI3K inhibitor. Cells in group IV were treated with 150 μmol/L of NMDA for 30 min followed by 10 μmol/L of baicalin and LY294002 (Calbiochem, La Jolla, CA, USA) for 24 h [24].

### Flow cytometry analysis

RGC-5 cells with different treatments were then digested, washed twice with ice-cold phosphate buffered saline (PBS), centrifuged for 5 minutes, and re-suspended in 200 μL of binding buffer. Next, cells were incubated with 10 μL Annexin V and 1 mg/mL propidium iodide (PI) (Beijing Biotech, China) in the dark at 20–25°C for 10 min. Apoptosis was quantified by flow cytometry (FACSCalibur; BD, New Jersey, USA) using Cell Quest Pro software (Beckman Coulter, Brea, CA, USA).

### Methyl thiazolyl tetrazolium (MTT) assay

Cell viability was assessed using the MTT assay. Briefly, RGC-5 cells were seeded into 96-well plates at a density of 2 × 10^5^ cells/well. RGC-5 cells with different treatments were incubated with MTT (0.5 mg/mL; Sigma-Aldrich) solution for 3 h. Medium was removed and dimethyl sulfoxide (DMSO; 200 μL) was added to each well. The optical density (OD) of each well was measured at a wavelength of 490 nm using a Multiskan Ascent Revelation Plate Reader (Thermo Fisher Scientific, Waltham, MA, USA) and the data are normalized to cell absorbance without NMDA stimulation.

### Measurement of reactive oxygen species (ROS)

Intracellular ROS accumulation was measured using 2,7-dichlorodihydrofluorescein diacetate (H2DCF-DA). Briefly, after different treatments, RGC-5 cells were washed and then stained with 10 μmol/L H2DCF-DA (Sigma-Aldrich) in serum-free medium for 30 minutes at 37°C in the dark. The cells were photographed using a fluorescence microscope (Olympus, Tokyo, Japan).

### Estimation of malondialdehyde (MDA)

MDA reacts with thiobarbituric acid to produce a fluorescent product that can be measured using a microplate reader (Thermo Fisher Scientific, Waltham, MA, USA) at a wavelength of 535 nm. After different treatments, RGC-5 cells were harvested, and MDA levels were determined using an MDA detection kit from Nanjing Jiancheng Bioengineering Institute (Nanjing, China) according to the manufacturer’s instructions.

### Animal grouping

C57BL/6 mice were divided into four groups (n = 10/group): control, model, Model+normal saline (NS), and Model+BAI (BAI refers to baicalin). Mice in the Model+BAI group were intraperitoneally injected with baicalin (50 mg/kg [23]) once a day for 15 days, and mice in the Model+NS group were intraperitoneally injected by identical doses of NS once a day for 15 days. Injections of baicalin or NS were performed after the successful establishment of the mouse model of glaucoma. Finally, all mice in different groups were killed by neck dislocation and the right eyeballs were stored in the refrigerator at −80°C for later experiments. All the animal experiments were approved by the Ethics Committee of Henan Provincial People’s Hospital (DWLL202110011; Henan, China).

### Establishment of a mouse model of glaucoma

C57BL/6 healthy mice (male, 8–10 weeks, weighing 25 g ± 2 g) were purchased from Jiangsu ALF Biotechnology (Nanjing, China). All mice were kept under the 12-h day/night cycle and fed with standardized pellet feed at 20°C. For this experiment, 30 mice were randomly divided into the glaucoma group, whereas the remaining 10 mice were in the control group (negative control). As previously described [25], episcleral venous occlusion with cauterization was used to establish a mouse model of glaucoma with chronic EIOP. Mice in the glaucoma group were intraperitoneally injected with 1% pentobarbital sodium (P3761, 50 mg/kg, Sigma Aldrich, USA) for general anesthesia and pentobarbital sodium was used to dropwise anesthetize their ocular surfaces. The bulbar conjunctiva at the limbus of the cornea was cut off in the supratemporal, supratemporal, and suprasal quadrants. After separation of the extraocular muscle and fascia, three proximal external scleral veins were burned with a sterile needle, resulting in congestion and whitening of the proximal and distal veins. The left eye of the mice served as the untreated control. After the surgery, the mixture of saline and gentamicin (G3632; Sigma-Aldrich Chemical Company, USA) was applied to wash the conjunctival sac. Erythromycin ophthalmic ointment (Chen Xin Pharmaceutical Co., Ltd., Beijing, China) was used to treat the eye area 3 times a day for 3 consecutive days to relieve inflammatory response. Next, the IOP of mice in the control group and glaucoma group was measured by Goldmann applanation tonometer (Shanghai Precision Scientific Instruments Co., Ltd., Shanghai, China) at 0, 3, 7, 14, 21, 28 and 42 days. After 42 days, the model is considered successfully built only when the average IOP is observed to remain above 22 mmHg (1 mmHg = 0.133 kPa).

### Hemotoxylin and eosin (H&E) staining

Eyes isolated from mice were fixed in 4% paraformaldehyde, dehydrated, paraffin-embedded, and sliced up. Next, the eye specimen was subjected to hematoxylin staining for 5 min and eosin staining for 2 min. After that, the stained eye specimen was washed in distilled water for 1 min. Gradient alcohol and xylene were used for dehydration and cleanout of the specimen. Morphological structure of eye specimen was observed via a light microscope (Olympus). Ganglion cell layer (GCL) thickness was quantified by the ImageJ software.

### Counting of RGCs

The right eyeballs of each group were thoroughly rinsed twice with 0.1 M PBS, and the anterior segments 1 mm away from corneoscleral limbus were removed. The vitreous bodies were clipped with toothless forceps, and the retinas were bluntly separated with tip‐curved forceps. The eyeballs were fixed in 4% polyoxymethylene for 30 min at room temperature, washed with PBS three times for 10 min, and put into 30% sucrose solution overnight in a refrigerator at 4°C. Furthermore, the eyeballs were washed with PBS three times for 10 min and blocked with 10% normal donkey serum overnight in a refrigerator at 4°C. After the addition of primary antibody against brain‐specific homeobox/POU domain protein 3B (dilution 1:1,000, sc‐514,474; Santa Cruz, CA), the eyeballs were put in a refrigerator overnight at 4°C for 6 days and washed with PBS three times for 10 min. With the addition of the Alexa Fluor® 488 labeled donkey anti‐mouse secondary antibody immunoglobulin G (dilution 1:50, ab150105; Abcam, Cambridge, MA), the eyeballs were placed overnight in a refrigerator at 4°C and washed with PBS for 10 min. With retinal stretched preparation and vitreous body upward, the retinas were mounted, stored without light, and put into a refrigerator overnight for drying at 4°C. Moreover, the retinas were divided into four quadrants with optic nervous papilla in the center and nasal and temporal direction as the ordinate. Each quadrant chose three areas from the optic disc of 1/6, 1/2, 5/6 retinal radius; photos were taken using a fluorescent microscope. Afterward, the total number of RGCs in each field was calculated.

### Western blotting

RIPA lysis buffer (Beyotime, Nanjing, China) was used to extract proteins from RGC-5 cells and retinal tissues of mice. BCA Protein Assay Kit (Thermo Fisher Scientific, USA) was applied to determine concentration of isolated proteins. Protein samples were loaded at 12% SDS-PAGE and then transferred onto PVDF membranes (Millipore, USA). Subsequently, the membranes were incubated with primary antibodies at 4°C overnight, which included anti-microtubule-associated protein 1 light chain 3B (LC3 B) (1:500; ab239416; Abcam), anti-Beclin-1 (1:500; ab210498; Abcam), anti-autophagy-related gene 5 (ATG5) (1:500; ab238092; Abcam), anti-phospho protein kinase B (pAKT) (1:500; ab38449; Abcam), anti-protein kinase B (AKT) (1:500; ab8805; Abcam), anti-phospho phosphoinositide 3-kinase (pPI3K) (p85, 1:1000; ab182651; Abcam), anti-phosphoinositide 3-kinase (PI3K) (p85; 1:1000; ab191606; Abcam) and β-actin (1:1000; ab8227; Abcam). LC3 is a ubiquitin-like molecule that is the mammalian homologue of the Atg8 encoded product in yeast [26,27]. LC3 has three isotypes including LC3A, LC3B, and LC3C, while only LC3B-II is correlated with increased levels of autophagic vesicles. After translation, the unprocessed form of LC3 is proteolytically cleaved by Atg4 protease and leads to formation of LC3-I with a carboxyterminal exposed glycine. When autophagy is activated, the exposed glycine of LC3-I is conjugated to the highly lipophilic phosphatidylethanolamine moiety to generate LC3-II, which is used as an autophagy marker [28,29]. Next, the membranes were incubated with secondary antibody horseradish peroxidase-labeled immunoglobulin G (ab6721; 1:1000; Abcam) at room temperature for 2 h. The protein bands were visualized by enhanced chemiluminescence reagent (Bio-Rad) and analyzed by the ImageJ software.

### Statistical analysis

All collective data were analyzed using SPSS version 21.0 statistics software (IBM Corp., Armonk, NY). The measurement data were expressed as the mean ± standard deviation. The differences between two groups were compared using *t* test and comparisons among multiple groups were analyzed by one‐way analysis of variance. If these tests were not used, the rank sum test was used. *P* < 0.05 was considered statistically significant.

## Results

### NMDA induces autophagy and injury of RGCs

**Figure 1.**
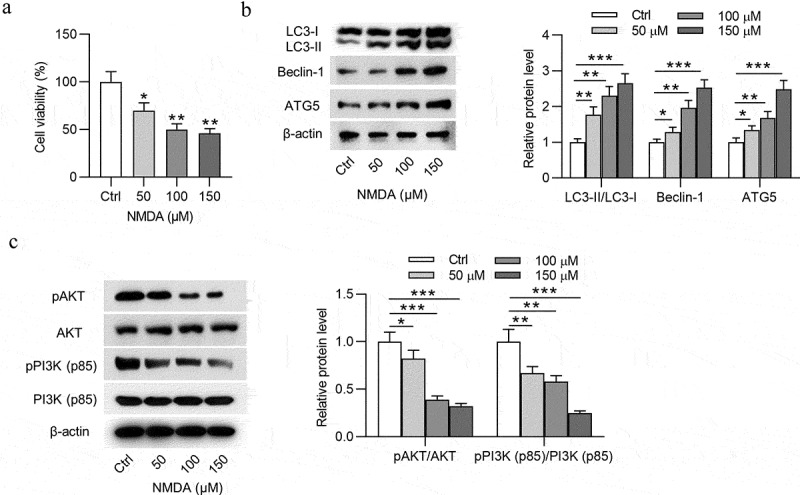
**NMDA induces autophagy and injury of RGCs**. (a) The viability of RGCs treated with different concentrations (0, 50, 100 or 150 μmol/L) of NMDA was evaluated by MTT assay. (b) The expression of autophagy-related proteins (LC3-I, LC3-II, Beclin-1 and ATG5) in RGCs treated with different concentrations (0, 50, 100 or 150 μmol/L) of NMDA was examined by Western blotting. (c) The expression of PI3K/AKT signaling-associated proteins (pAKT, AKT, pPI3K and PI3K) in RGCs treated with different concentrations (0, 50, 100 or 150 μmol/L) of NMDA was tested by Western blotting. *p < 0.05, ** *p* < 0.01, ***p < 0.001

We first explored the autophagy and PI3K pathways in RGCs by NMDA treatment. NMDA with different concentrations (0, 50, 100, or 150 μmol/L) was used to treat RGCs. According to MTT assay results, the viability of RGCs was gradually decreased with NMDA concentration increasing (Figure 1a). Subsequently, the expression of autophagy-related proteins (LC3-I, LC3-II, Beclin-1, and ATG5) and PI3K/AKT signaling-associated proteins (pAKT, AKT, pPI3K, and PI3K) in RGCs were detected by Western blotting. As Figure 1b demonstrated, the protein expression of LC3-II, Beclin-1, and ATG5 in RGCs was increased with NMDA concentration increasing, suggesting that NMDA induces autophagy of RGCs. Furthermore, the protein expression of p-AKT and p-PI3K in RGCs was declined with the increased concentration of NMDA (Figure 1c), which indicated that NMDA inhibited the PI3K/AKT signaling in RGCs. All these results revealed that 150 μmol/L of NMDA has the best effects on inducing cell injury, autophagy, and inactivating PI3K/AKT signaling in RGCs. Thus, 150 μmol/L of NMDA was used in the following experiments.

### Baicalin inhibits NMDA-induced autophagy, cell injury and oxidative stress of RGCs via activating PI3K/AKT signaling

**Figure 2.**
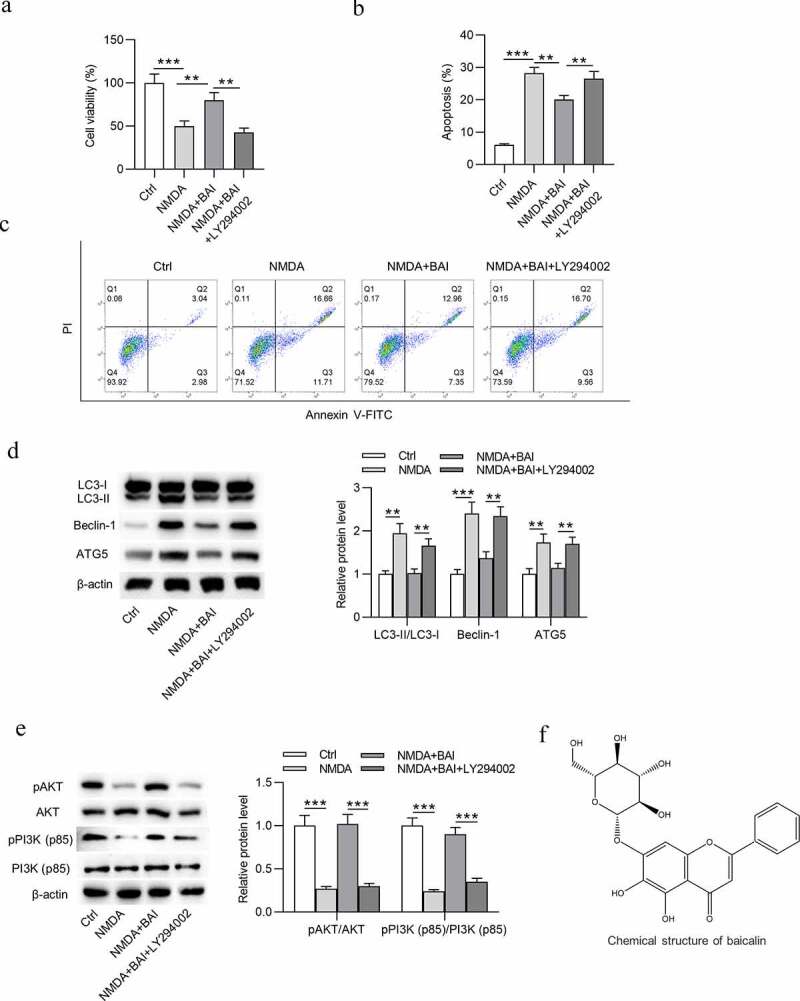
**Baicalin inhibits autophagy and injury of NMDA-treated RGCs via activating PI3K/AKT signaling**. (a) The viability of RGCs in four groups (Ctrl, NMDA, NMDA+BAI and NMDA+BAI+LY294002) was assessed by MTT assay. (b-c) Apoptosis of RGCs in four groups (Ctrl, NMDA, NMDA+BAI and NMDA+BAI+LY294002) was tested by flow cytometry. (d-e) The expression of autophagy-related proteins (LC3-I, LC3-II, Beclin-1 and ATG5) and PI3K/AKT signaling-associated proteins (pAKT, AKT, pPI3K and PI3K) in RGCs in four groups (Ctrl, NMDA, NMDA+BAI and NMDA+BAI+LY294002) was evaluated by Western blotting. (f) Chemical structure of baicalin was presented. ** *p* < 0.01, *** *p* < 0.001

**Figure 3.**
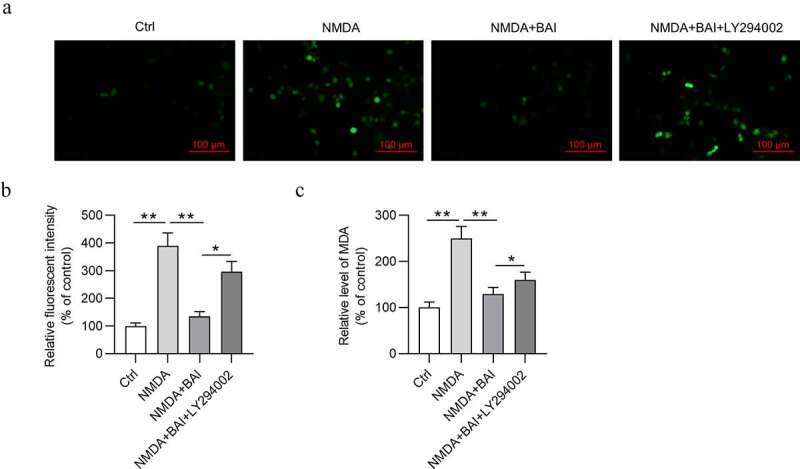
**Baicalin restrains NMDA-induced oxidative stress injury of RGCs via activating PI3K/AKT signaling**. (a) DCFH-DA staining assay was performed and (b) ROS and (c) MDA levels in RGCs in four groups (Ctrl, NMDA, NMDA+BAI and NMDA+BAI+LY294002) were measured. *p < 0.05, ** *p* < 0.01

Impacts of NMDA on the apoptosis, autophagy, and oxidative stress of RGCs were subsequently detected. To mimic glaucoma environment *in vitro*, NMDA at the concentration of 150 μmol/L was used to treat RGCs. After NMDA stimulation, the viability of RGCs was reduced (Figure 2a) and apoptosis rate of RGCs was increased (Figure 2(b,c)). In addition, NMDA treatment increased the protein expression of LC3-II, Beclin-1, and ATG5 (Figure 2d) and decreased the protein expression of p-AKT and p-PI3K (Figure 2e) in RGCs. As Figure 3(a-c) showed, NMDA elevated ROS and MDA levels in RGCs (Figure 3(a-c)), indicating that NMDA induced oxidative stress of RGCs. All these findings meant that a cellular model of glaucoma was established successfully. Next, to figure out the role of baicalin in glaucoma *in vitro*, baicalin was used to treat RGCs after NMDA stimulation. MTT assay revealed that baicalin offset the NMDA-induced decrease in viability of RGCs (Figure 2a). Flow cytometry analysis demonstrated that baicalin attenuated the promotive effects of NMDA on apoptosis of RGCs (Figure 2b-c). The elevation in LC3-II, Beclin-1, and ATG5 protein levels and decrease in p-AKT and p-PI3K protein levels were caused by NMDA in RGCs and were counteracted by baicalin (Figure 2d-e). The chemical structure of baicalin (5,6-dihydroxy7-O-glucuronide) is shown in figure 2f. DCFH-DA staining assay suggested that the increase of ROS and MDA levels in NMDA-treated RGCs was neutralized by baicalin (Figure 3a-c). Next, to explore whether baicalin can regulate the PI3K/AKT signaling in NMDA-stimulated RGCs, LY294002 was applied to treat RGCs. However, LY294002 treatment was found to reverse all effects of baicalin on cell viability (Figure 2a), cell apoptosis (Figure 2b-c), autophagy-related protein expression (Figure 2d), PI3K/AKT signaling-associated protein expression (Figure 2e), and ROS and MDA levels (Figure 3a-c) in NMDA-treated RGCs. To sum up, baicalin inhibits autophagy, cell injury and oxidative stress of NMDA-treated RGCs via activating PI3K/AKT signaling.

### Successful establishment of a mouse model of glaucoma

**Figure 4.**
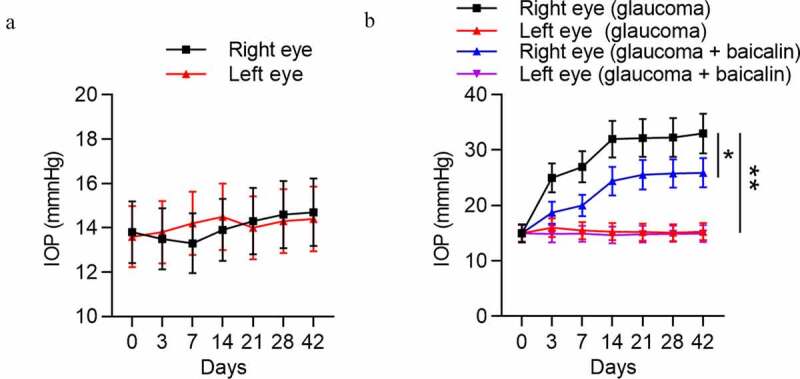
**Successful establishment of a mouse model of glaucoma**. (a) IOP variation in left eye and right eye of mice in the control group (n = 10) was measured at 0, 3, 7, 17, 21, 28 and 42 days. (b) IOP variation in left eye and right eye of mice with glaucoma after baicalin treatment was measured at 0, 3, 7, 17, 21, 28 and 42 days. ** *p* < 0.01

IOP variation of both eyes of mice in the control group, glaucoma group, and glaucoma+baicalin group was measured. As Figure 4a demonstrated, there was no obvious difference between IOP of right eye and IOP of left eye in the control group (*p* > 0.05). However, a significant upregulation was detected in IOP of right eye compared with IOP of left eye in the experimental group (*p* < 0.01). Simultaneously, IOP of right eye in the experimental group was gradually increased with time increasing, while no obvious IOP variation was detected in left eye and right eye of the control group during experimental time. Moreover, baicalin treatment induced the lower IOP of the right eyes in glaucoma mice (Figure 4b). All these data suggested that a mouse model of glaucoma was established successfully, and baicalin can alleviate glaucoma.

### Baicalin increases RGC number and attenuates pathological changes of glaucoma mice

**Figure 5.**
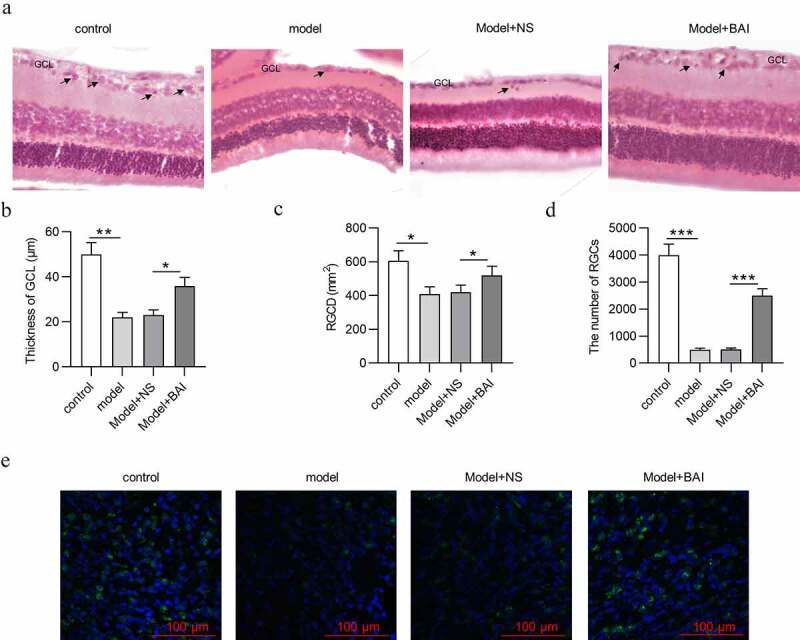
**Baicalin increases RGC number and attenuates pathological changes in retinas of glaucoma mice**. (a) The structure and morphology of retina of mice in four groups (control, model, Model+NS and Model+BAI) were presented by H&E staining (scale bar = 25 μm). (b) Thickness of GCL and (c) RGCD of mice in four groups (control, model, Model+NS and Model+BAI) were measured. (d) The number of RGCs in retina tissues of four groups (control, model, Model+NS and Model+BAI) was counted. (e) Counting of RGCs using immunoflurescence staining of brain specific homeobox/POU domain protein 3B (Brn3b) in retina tissues of four groups (control, model, Model+NS and Model+BAI). *p < 0.05, ** *p* < 0.01, *** *p* < 0.001

After mice were successfully induced with glaucoma, the pathological changes of retinal tissues in different groups (control, model, Model+NS and Model+BAI) were detected by H&E staining. As Figure 5a-c indicated, control group exhibited clear retinal layer and compact and neatly arranged RGCs with higher density. However, the structure and morphology of retinal tissues in glaucoma mice changed with indistinct layer of retinas, decrease in thickness of GCL and RGC density (RGCD) (Figure 5a-c). In addition, the number of RGCs in retinal tissues was decreased in model group compared with control group (Figure 5d-e). All these findings further confirmed successful establishment of a mouse model of glaucoma. To explore the effects of baicalin in glaucoma mice, baicalin were intraperitoneally injected into the glaucoma mice. Compared with Model+NS group, baicalin administration mitigated pathological changes of retinal tissues in glaucoma mice, evidenced by increase in thickness of GCL and RGCD in Model+BAI group (Figure 5a-e). Furthermore, reduction in RGC number in Model+NS group was attenuated by baicalin treatment in glaucoma mice (Figure 5d-e). All in all, baicalin attenuates glaucoma pathological changes and increases the number of RGCs in EIOP-stimulated mice.

### Baicalin inhibits autophagy and activates PI3K/AKT signaling in glaucoma mice

**Figure 6.**
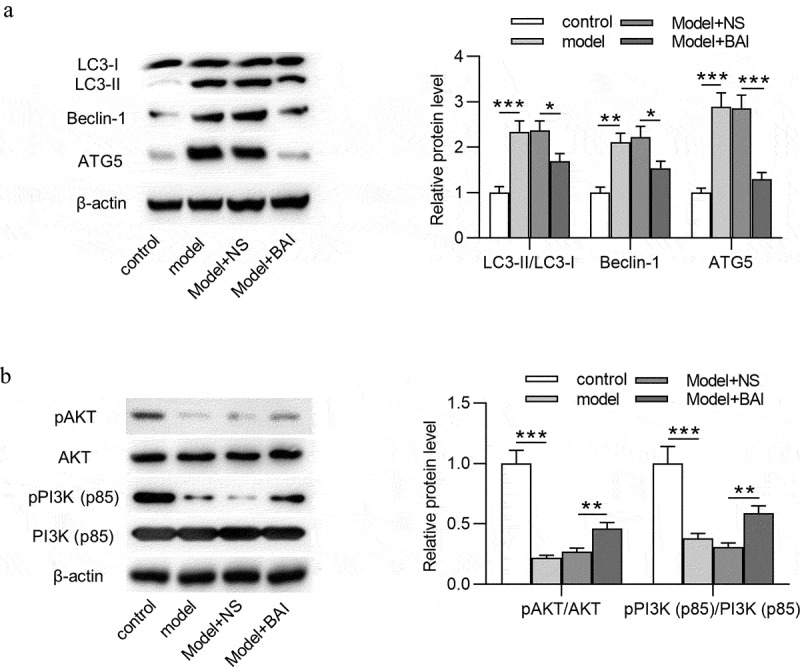
**Baicalin inhibits autophagy and activates PI3K/AKT signaling in glaucoma mice**. (a) The expression of autophagy-related proteins (LC3-I, LC3-II, Beclin-1 and ATG5) and (b) PI3K/AKT signaling-associated proteins (pAKT, AKT, pPI3K and PI3K) in retinal tissues of four groups (control, model, Model+NS and Model+BAI) was detected by Western blotting. **p < 0.01, *** *p* < 0.001

To investigate the regulatory role of baicalin in autophagy and PI3K/AKT signaling in glaucoma mice, the expression of autophagy-related proteins (LC3-I, LC3-II, Beclin-1, and ATG5) and PI3K/AKT signaling-associated proteins (pAKT, AKT, pPI3K and PI3K) in retinal tissues of four groups (control, model, Model+NS and Model+BAI) was detected by Western blotting. As Figure 6a-b showed, increased protein expression of LC3-II, Beclin-1, and ATG5 and decreased protein expression of p-AKT and p-PI3K was observed in model group compared with control group, suggesting that autophagy was promoted and PI3K/AKT signaling was inactivated in glaucoma mice. However, baicalin treatment attenuated the EIOP-induced increase in protein expression of LC3-II, Beclin-1, and ATG5 in Model+NS group, which indicated that baicalin inhibited autophagy in glaucoma progression (Figure 6a). Additionally, baicalin administration reversed the EIOP-induced decrease in p-AKT and p-PI3K protein levels in Model+NS group, meaning that baicalin activated PI3K/AKT signaling in glaucoma (Figure 6b). To sum up, baicalin inhibits autophagy and activates PI3K/AKT signaling in glaucoma pathogenesis.

## Discussion

NMDA is an important excitatory neurotransmitter L-glutamate homologue in the mammalian central nervous system [30]. Accumulating studies have revealed that NMDA can induce RGC degradation and retinal injury [31,32]. In the present study, NMDA with different concentrations (0, 50, 100, or 150 μmol/L) was used to treat RGCs. Viability of RGCs was gradually decreased with NMDA concentration increasing. As a self-digestion mechanism of cells, autophagy can degrade damaged organelles and proteins in response to cell metabolic stress and also can cause cell death [33]. Elevation in autophagy-related proteins was detected in NMDA-treated RGCs or rats, and RGC autophagy contributed to retinal neuron loss in the ganglion cell layer [34]. Herein, the protein expression of autophagy-related proteins (LC3-II, Beclin-1 and ATG5) in RGCs was concentration-dependently increased by NMDA, suggesting that NMDA induces autophagy of RGCs.

PI3K (phosphatidylinositol kinase) is a dimer composed of the regulatory subunit p85 and the catalytic subunit p110 [35]. Multiple evidence has identified that the PI3K/AKT signaling restrains apoptosis and inflammatory response of RGCs in glaucoma development [36,37]. Moreover, increased ROS during oxidative stress can induce autophagy [38]. The PI3K/AKT signaling negatively regulates the oxidative stress response [39] and the autophagy pathway [40]. In this study, the protein expression of p-AKT and p-PI3K in RGCs was decreased by NMDA, meaning that NMDA inhibits the PI3K/AKT signaling in RGCs.

Baicalin was revealed to play a neuroprotective role in glaucoma progression [23,41]. Our findings revealed that baicalin exerted pro-proliferative and anti-apoptotic effects on NMDA-treated RGCs, suggesting that baicalin mitigates NMDA-induced cell injury of RGCs. Baicalin has been reported to regulate autophagy via PI3K/AKT signaling pathway [42,43]. In this study, NMDA-induced the elevation in levels of LC3-II, Beclin-1, and ATG5 in RGCs was counteracted by baicalin, which indicated that baicalin inhibits autophagy of NMDA-treated RGCs. In addition, baicalin reversed the NMDA-induced inhibition in p-AKT and p-PI3K protein levels in RGCs, demonstrating that baicalin activates PI3K/AKT signaling in NMDA-stimulated RGCs. As a key step in glaucoma pathophysiology, oxidative stress is connected to RGC apoptosis and outflow facility dysregulation [44,45]. Our findings showed that the NMDA-induced increase of ROS and MDA levels in RGCs was neutralized by baicalin, indicating that baicalin inhibits oxidative stress in glaucoma *in vitro*. Next, LY294002 treatment counteracted the effects of baicalin on cell viability, apoptosis, autophagy, ROS, and MDA levels in NMDA-treated RGCs. It can be concluded that baicalin restrains cell injury, autophagy, and oxidative stress via activating PI3K/AKT signaling in an *in vitro* model of glaucoma. The *in vivo* data revealed that baicalin offset the decrease in GCL thickness and RGCD in retinas of glaucoma mice. Moreover, the number of RGCs was significantly decreased in glaucoma mice, and then increased by baicalin treatment. Similarly, baicalin also inhibited autophagy and activated PI3K/AKT signaling *in vivo*. Therefore, baicalin suppresses glaucoma pathogenesis by regulating PI3K/AKT signaling *in vitro* and *in vivo*.

Since other Chinese herb medicines, such as *lycium barbarum* [46], *cordyceps cicadae mycelia* [47] and *resveratrol* [48] were reported to alleviate glaucoma, more studies about whether these Chinese herb medicines can regulate PI3K/AKT signaling in glaucoma development should be conducted. In addition to PI3K/AKT signaling, other signal pathways including mitogen-activated protein kinase 1 signaling [49] and nuclear factor kappa B signaling [50], were demonstrated to be modulated by baicalin and were not mentioned in the present study, which is a limitation of our study. Studies on whether baicalin can regulate these signaling pathways in glaucoma pathogenesis also should be performed in the future. Moreover, the methods to induce the *in vivo* and *in vitro* models of glaucoma were not consistent, and we will use the NMDA-induced glaucoma mice to explore the influences of baicalin on autophagy and PI3K pathways in glaucoma if necessary.

## Conclusion

Baicalin restrained the NMDA-induced cell apoptosis, autophagy, and oxidative stress of RGCs by activating the PI3K/AKT signaling *in vitro. In vivo* studies showed that baicalin activated PI3K/AKT signaling to suppress autophagy, subsequently attenuating the pathological changes in retinal tissues of glaucoma mice. These findings indicated baicalin as a promising drug for glaucoma treatment.

## Data Availability

The datasets used during the current study are available from the corresponding author on reasonable request.
